# Peri-operative Antibiotic Prophylaxis in K-Wire Fixation: A Systematic Review and Meta-analysis

**DOI:** 10.1007/s43465-023-00879-6

**Published:** 2023-04-08

**Authors:** Ahmad Abul, Mohammad Karam, Shaima Al-Shammari, Peter Giannoudis, Hermant Pandit, Sohail Nisar

**Affiliations:** 1 Division of Surgical and Interventional Sciences, University College London, Gower Street, London, WC1E 6BT UK; 2 Department of Surgery, Farwaniya Hospital, Kuwait City, Kuwait; 3 Leeds Orthopaedic Trauma Sciences, Leeds University, Leeds, UK; 4 Leeds Teaching Hospitals NHS Trust, Beckett Street, Leeds, LS9 7TF UK; 5 Leeds Institute of Rheumatic and Muskuloskeletal Medicine, Chapel Allerton, Leeds, UK

**Keywords:** K-wires, Antibiotic prophylaxis, Infection rate

## Abstract

**Introduction:**

There are currently no standardised guidelines on whether antibiotic prophylaxis is required for Kirschner wire (K-wire) fixation to minimise the risk of surgical site infection when used in patients undergoing clean orthopaedic surgery.

**Purpose:**

To compare the outcomes of antibiotic prophylaxis versus no antibiotic in K-wire fixation when used in either in trauma or elective orthopaedics.

**Methods:**

A systematic review and meta-analysis were performed as per the Preferred Reporting Items for Systematic Reviews and Meta-analyses (PRISMA) Guidelines and a search of electronic information was conducted to identify all randomised controlled trials (RCTs) and non-randomised studies comparing the outcomes of antibiotic prophylaxis group versus those without antibiotic in patients undergoing orthopaedic surgery in which K-wire fixation was used. Incidence of surgical site infection (SSI) was the primary outcome. Random effects modelling was used for the analysis.

**Results:**

Four retrospective cohort studies and one RCT were identified with a total of 2316 patients. There was no significant difference between the prophylactic antibiotic and no antibiotic groups in terms of incidence of SSI (odds ratio [OR] = 0.72, *P* = 0.18).

**Conclusions:**

There is no significant difference in administering peri-operative antibiotics for patients undergoing orthopaedic surgery using K-wire.

## Introduction

Kirschner (K) wires are commonly used in orthopaedic surgery for fixation of unstable fractures such as supracondylar humerus fractures in paediatric patients [1], distal radius fractures in paediatric and adult patients [2] and elective surgery of the foot. They can be inserted percutaneously with minimal damage to soft tissues such as tendons and neurovascular structures, followed by cutting and bending of the wires [3, 4]. The wires are then either left protruding outside the skin or buried. The limb is immobilised with either a splint or cast [5, 6].

There are associated complications related to the use of K-wire; one being pin-tract infections with an incidence rate ranging between 1.9 and 34% in hand and wrist fractures [5]. Superficial infections may respond to regular change in dressing or a course of oral antibiotics. In more severe cases pin removal may be required which can be problematic if the fracture has not united. More serious infections including osteomyelitis may require hospital admission for intravenous antibiotics or a further operation [2, 5].

While there are published guidelines relating to the use of peri-operative antibiotics for orthopaedic surgery, currently, there is no specific guidance for surgery using K-wire. The National Institute for Health and Care Excellence (NICE) guidelines recommends the use of antibiotic prophylaxis before operations that include clean wounds with prosthesis or implant insertion as well as contaminated and clean-contaminated surgical incisions [7]. Additionally, Scottish Intercollegiate Guidelines Network (SIGN) recommend antibiotic prophylaxis to be administered in orthopaedic surgery if the procedure involves an insertion of a prosthetic device [8]. The guidelines also recommend prophylactic antibiotics whilst carrying out an open reduction of a closed fracture. However, these guidelines do not specify whether this recommendation is applicable for percutaneously inserted K-wire for closed fractures.

Some authors have suggested the use of post-operative antibiotics; however, this is not routine practice in the UK [9]. The use of prophylactic antibiotics post-operatively has been explored in previous studies. A retrospective study including 618 patients has shown that post-operative antibiotics did not lower the incidence of surgical site infection (SSI): 1.7% with antibiotic use compared to 1.8% for no antibiotic group [9].

Interestingly, with regards to peri-operative antibiotic use some authors have argued there is a low overall SSI risk following K-wire fixation; therefore, peri-operative antibiotics are not indicated [10]. However, in other studies patients who were not given peri-operative prophylaxis had a higher rate of post-operative infection [11].

Overall, there is no consensus or clear guidance on the administration of antibiotics for K-wire. Reducing the incidence of SSI needs to be balanced with potential risks of the unnecessary use of antibiotics, such as antibiotic resistance and allergic reaction. Currently, there are no systematic reviews or meta-analyses that investigate the use of prophylactic antibiotics for orthopaedic surgery using K-wire. The aim of the study therefore is to investigate incidence of SSI using prophylactic antibiotics compared to no antibiotics in the context of percutaneous fixation of fractures with K-wire.

## Methods

A systematic review and meta-analysis was conducted as per the Preferred Reporting Items for Systematic Reviews and Meta-Analyses (PRISMA) guidelines [12].

### Eligibility Criteria

All randomised control trials and observational studies investigating the use of a prophylactic antibiotics group with a comparator for patients with fractures of the upper (e.g. supracondylar fractures of the humerus, distal radius fracture) or lower extremity (e.g. toe fractures) requiring K-wire fixation were eligible for inclusion. The group without prophylactic antibiotic was the intervention group of interest and the group with prophylactic antibiotic was the comparator. No age, gender or morbidity status restrictions were used but for language, restrictions were set to English only. Cases reports and observational studies with no comparator were excluded.

### Outcome Measures

Incidence of SSI is the primary outcome which was assessed between the two groups. No secondary outcomes were identified.

### Literature Search Strategy

Two authors M.K. and A.A independently searched the following electronic databases: MEDLINE, EMBASE, EMCARE, CINAHL, and the Cochrane Central Register of Controlled Trials (CENTRAL). The last search was run on 10th January 2022. Thesaurus headings, search operators, and limits in each of the above databases were adapted accordingly. In addition, World Health Organization International Clinical Trials Registry (http://apps.who.int/trialsearch/), ClinicalTrials.gov (http://clinical-trials.gov/), and ISRCTN Register (http://www.isrctn.com/) were searched for details of ongoing and unpublished studies. No language restrictions were applied in our search strategies. The search terminologies included: “Kirschner wires”, “K-wires”, "Percutaneous wires", “Antibiotic Prophylaxis”, “Wound Infection” and “Surgical Site Infection”. The bibliographic lists of relevant articles were also reviewed.

### Selection of Studies

The title and abstract of articles identified from the literature searches were assessed independently by two authors (A.A. and M.K.). The full texts of relevant reports were retrieved and those articles that met the eligibility criteria of our review were selected. Any discrepancies in study selection were resolved by discussion between the authors.

### Data Extraction and Management

An electronic data extraction spreadsheet was created in line with Cochrane’s data collection form for intervention reviews. The spreadsheet was pilot tested in randomly selected articles and adjusted accordingly. Our data extraction spreadsheet included study-related data (first author, year of publication, country of origin of the corresponding author, journal in which the study was published, study design, study size, clinical condition of the study participants, type of intervention, and comparison), baseline demographics of the included populations (age and gender) and primary outcome data. The authors A.A. and M.K. cooperatively collected and recorded the results and any disagreements were solved via discussion.

### Data Synthesis

Data synthesis was conducted using the Review Manager 5.3 software. The extracted data was entered into Review Manager by two independent authors (A.A. and M.K.). The analysis used was based on the random effect model. The results were reported in forest plots with 95% confidence intervals (CIs).

For dichotomous outcomes, the odds ratio (OR) was calculated between the two groups. The OR is the risk of an event in the group receiving prophylactic antibiotic compared with the group without antibiotic. An OR of  > 1 for the SSI would favour the prophylactic antibiotic group, an OR of  < 1 would favour the group without antibiotic and an OR of 1 would favour neither group.

### Assessment of Heterogeneity

Heterogeneity among the studies was assessed using the Cochran *Q* test (*χ*^2^). Inconsistency was quantified by calculating *I*^2^ and interpreted using the following guide: 0–25% represents low heterogeneity, 25–75% moderate heterogeneity, and 75–100% high heterogeneity.

### Methodological Quality and Risk of Bias Assessment

The Cochrane Collaboration’s Tool was used to assess the quality of the RCTs included in the study (Table 2). For non-randomised studies, the Newcastle–Ottawa scale [13] was used to assess its quality which offers a star system for analysis (Table 3). It offers a maximum score of nine stars across three domains including selection, comparability and exposure. It offers a maximum score of nine stars across three domains including selection, comparability and exposure. The overall rating of either good, fair or poor quality was based on the Agency for Healthcare Research and Quality (AHRQ) standards [13]. This was assessed by two authors (M.K and A.A) and a third author; S.A.S was used as an adjudicator if consensus was required.

## Results

### Literature Search Results

**Fig. 1:**
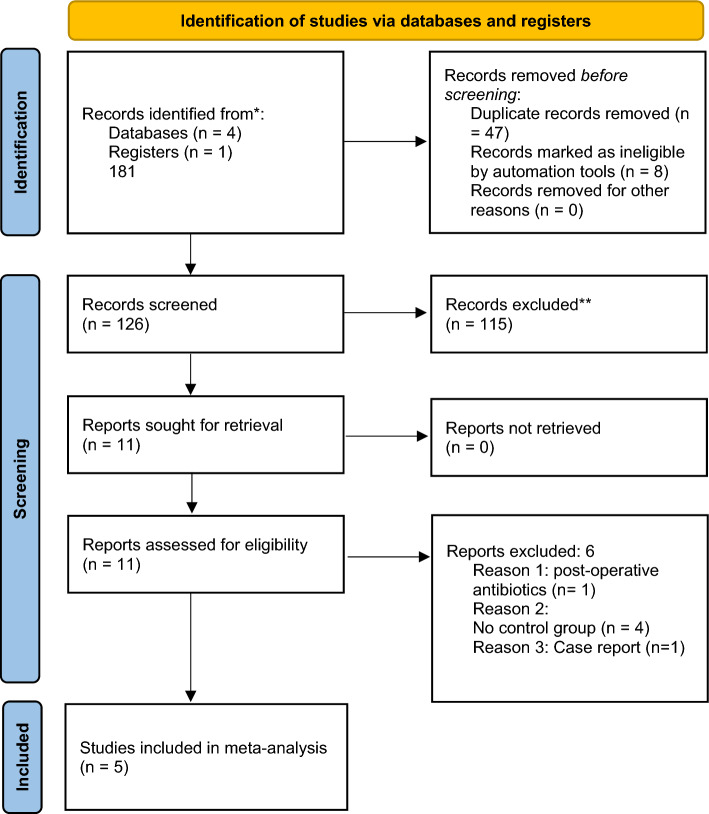
PRISMA flow diagram. The PRISMA diagram details the search and selection processes applied during the overview. PRISMA, Preferred Reporting Items for Systematic Reviews and Meta-Analyses

Our search strategy retrieved 181 studies and after a thorough screening of the retrieved articles, the authors identified five studies [5, 6, 14–16] in total which met the eligibility criteria (Fig. 1).

### Description of Studies

Baseline characteristics of the included studies are summarised in Table 1. This includes the percentage SSI of the cohort for each of these studies.

**Table 1:** (a) Baseline characteristics of the included studies 1. (b) Baseline characteristics of the included studies 2 (continued)

a						
Study (year)	Journal, country	Study design	Mean age (yrs)	Gender (male:female)	Co-morbidities	Total study sample (Abx: no Abx)
Heras et al. (2005) [14]	Clinical Orthopaedics and Related Research, Spain	Retrospective review	6.7 (SD NR)	56:21	NR	77 (46:31)
Lobst et al. (2007) [6]	Journal of Paediatric Orthopaedics, USA	Retrospective review	5.4 (SD NR)	162:142	NR	304 (97:207)
Bashyal et al. (2009) [15]	Journal of Paediatric Orthopaedics, USA	Retrospective cohort study	NR	NR	NR	622 (163:459)
van Leeuwen et al. (2016) [5]	Journal of Hand Surgery, USA	Retrospective review	42 (SD NR)	748:465	Diabetes: 88 (9 infected vs 79 no infection)	1213 (981:232)
Mangwani et al. (2017) [16]	Journal of Foot and Ankle Surgery, UK	RCT	Antibiotic: 58 ± 17.5 No Antibiotic: 62.7 ± 14.7	NR	Diabetes: 6 4 had antibiotics given 2 didn’t have Antibiotic	100 (48:52)

b						
Study (year)	Population and treatment	Injury classification (*n*)	Buried/exposed K-wire (*n*)	Duration of K-wire	Prophylactic antibiotic (*n*)	Type of skin preparation used
Heras et al. (2005) [14]	Paediatric patients with displaced supracondylar fractures of the humerus treated with with closed reduction and percutaneous fixation with two lateral K-wire	Gartland Type II (39) Gartland type III (38)	Exposed (all)	4 weeks	Pre-operative IV antibiotic treatment of 50 mg/kg/day of cefazolin, divided into three doses	NR
Lobst et al. (2007) [6]	Paediatric patients with supracondylar humerus fractures treated with closed reduction and percutaneous pinning	Gartland Type II (125) Gartland Type III (179) Gustilo and Anderson Type I open fractures (3)	Exposed (all)	4–5 weeks	Single dose of peri-operatively IV antibiotics: cefazolin unless allergy required alternative option	Limited preparation and draping technique (semisterile technique): Betadine preparation of affected extremities and anticipated area of pin placement only. No drapes used
Bashyal et al. (2009) [15]	Paediatric patients with displaced or angulated supracondylar distal humerus fractures treated with the closed reduction and percutaneous pinning procedure	Gartland Type II (294) Gartland Type III (311)	Exposed (591) Buried (31)	3 weeks	Pre-operative IV antibiotics: Cefazolin (160), Clindamycin (2) or Vancomycin (1)	One of four options depending on surgeon: 1. Simple betadine preparation and towel draping 2. Betadine paint and towel draping 3. Spray betadine preparation and towel draping 4. Alcohol wash then betadine spray and towel draping
van Leeuwen et al. (2016) [5]	Adult patients undergoing percutaneous pinning in hand and wrist fractures	NR	Exposed (all)	32 days (mean)	NR	NR
Mangwani et al. (2017) [16]	Adult patients undergoing toe fusion surgery that required K-wire	NR	Buried in situ (all)	4–6 weeks	Flucloxacillin or teicoplanin	NR

*NR* not reported

### Primary Outcome(s)

#### Incidence of SSI

**Fig. 2:**
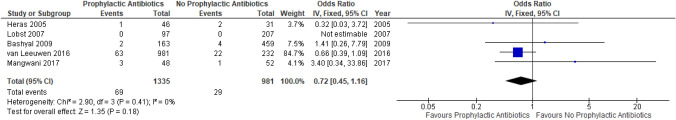
Forest plot of antibiotic group vs. no antibiotic group – incidence of SSI. Quantitative analysis showing the odds ratio in incidence of SSI reported by Heras et al. [14], Lobst et al. [6], Bashyal et al. [15], van Leeuwen et al. [5] and Mangwani et al. [16]

The incidence of SSI was reported in four studies, enrolling a total of 2316 patients (Fig. 2). There was no significant difference between the prophylactic antibiotic and no antibiotic groups in terms of incidence of SSI (odds ratio (OR) = 0.72, confidence interval (CI) = 0.45–1.16, *P* = 0.16). A low level of heterogeneity was found amongst the studies (*I*^2^ = 0%, *P* = 0.42).

### Methodological Quality and Risk of Bias Assessment

The Cochrane Collaboration’s Tool was used to assess the quality of the Mangwani et al. [16] showing an overall low risk of bias (Table 2). For non-randomised studies, the Newcastle–Ottawa Scale [13] was used to assess the quality of the four non-randomised studies (Table 3), which offers a star system for analysis. All four studies demonstrated a high selection and exposure but a low comparability score [5, 6, 13, 14]. Overall, all studies, except for van Leeuwen [5], were of good quality based on the AHRQ standards [13].

**Table 2:** Assessment of risk of bias of the randomised trials using the Cochrane collaboration’s tool

First author	Bias	Authors’ judgement	Support for judgement
Mangwani et al. [16]	Random sequence generation (selection bias)	Low risk	Stratified randomisation technique used
	Allocation concealment (selection bias)	Unclear risk	No information given
	Blinding of participants and personnel (performance bias)	Low risk	No blinding of participants, but unlikely to influence outcome (presence of infection)
	Blinding of outcome assessment (detection bias)	Low risk	No blinding but unlikely to influence outcome (presence of infection). All suspected infections were graded as per the modified Oppenheim’s classification
	Incomplete outcome data (attrition bias)	Low risk	Complete and consistent outcome data for all participants available- no data was missing
	Selective reporting (reporting bias)	Low risk	All outcome data reported under “Results”
	Other bias	Low risk	None were identified

**Table 3:** Assessment of risk of bias of retrospective cohort studies using Newcastle–Ottawa Scale. Overall, all studies except for van Leeuwen were of good quality according to AHRQ standards. Van Leeuwen had poor quality due to no mention of follow-up

Study	Selection	Comparability	Exposure
Heras et al. [14]	***	*	**
Lobst et al. [6]	***	*	***
Bashyal et al. [15]	****	*	***
van Leeuwen et al. [5]	***	*	*

## Discussion

This study demonstrated that there was no significant difference (OR 0.72, *P* = 0.18) in the incidence of reported SSI in patients undergoing K-wire fixation who receive prophylactic antibiotics when compared to patients who did not receive antibiotic prophylaxis. However, there is a slight trend that is in favour towards patients who received prophylactic antibiotics as seen in Fig. 1.

Cohort studies without comparators support this finding, in that the incidence of SSI reported across studies is similar whether antibiotics were used or not. For example, Schroeder et al. reported an SSI of 1.8% (11/618) in their cohort study in which all infected cases received pre-operative antibiotic [9]. Subramanian et al. reported on 100 patients (173 unburied K-wires) inserted without receiving prophylactic antibiotics with only 2% infection rate [17]. Studies concentrating on post-operative antibiotics found no significant link in the use of antibiotic therapy and the development of SSI. [9, 18].

Previous studies have elaborated on the importance of mechanism of action for antibiotic prophylaxis used in surgery [19]. The antibiotics used are bactericidal meaning they kill rather than prevent further growth of bacteria. However, concentrations of antibiotics seem to affect their properties, for example clindamycin being bacteriostatic at lower concentrations and bactericidal at higher concentrations [20]. Although in our study, peri-operative antibiotics are seen to be ineffective in reducing infection rate, other elective procedures such as hip surgery have much better success rates in the use of peri-operative antibiotics [21] which may be related to concentrations of antibiotics used as well as local antibiotics (in cement etc.).

Resistance to antimicrobials is becoming increasingly prevalent [2] which raises concerns when unnecessary antibiotics are used routinely. Routine administration of antibiotics has also been linked to other complications, for example, enterotoxaemia due to *Clostridium difficile* and the development of adverse reactions (22). Reduction of routine administration of antimicrobials may be cost efficient due to the overall reduction in healthcare costs [23]. It is therefore imperative to weigh the advantages and disadvantages of the prophylactic use of antibiotics.

A systematic approach was used in this review to provide a summary of the best available evidence and to assess the risk of bias of relevant retrospective studies [5, 6, 14–16]. With regard to the between-study heterogeneity, it was low (*I*^2^ = 0%). Based on the design and included population, the one RCT [16] and four non-randomised studies [5, 6, 13, 14] were standardised. All of these would make the conclusions of this study robust from the best available evidence.

Nevertheless, the reported outcomes of the current review should be studied in the context of inherent limitations. Only five studies were identified enrolling 2316 participants, which may not be sufficient for definite conclusions, exposing the findings to a potential type two error. Additionally, due to the limited number of studies included and the data available, it was not possible to perform a sensitivity analysis to separate the RCT study from the observational studies and which further weakens our analysis. The gap in sample sizes between the individual studies may have affected the analysis, such as van Leeuwen et al. [5] with a sample of 1213 participants compared to Heras et al. [14] with a sample of only 77 subjects. Additionally, Mangwani et al. [16] was the only RCT found to be included in the study which might limit the overall conclusions due to high selection bias of the retrospective studies. Furthermore, different studies had different approaches in K-wire fixation (either buried or exposed) which could have affected infection rates.

Noteworthy, a survey of Orthopaedic surgeons demonstrated a lack of consensus in routine practice [24]. Furthermore, no clear guidelines are available from NICE or SIGN [7, 8].

In conclusion, based on the current available evidence there is no significant difference in administering peri-operative antibiotics for patients undergoing fracture fixation with K-wire. Further high quality, prospective studies are required to improve the evidence base to influence guidelines.

## Data Availability

The datasets generated and analysed during the current study are available from the corresponding author on reasonable request.
